# Micro-/nanobubble oxygenation irrigation enhances soil phosphorus availability and yield by altering soil bacterial community abundance and core microbial populations

**DOI:** 10.3389/fpls.2024.1497952

**Published:** 2025-02-10

**Authors:** Qingyong Bian, Zhiduo Dong, Yupeng Zhao, Yaozu Feng, Yanbo Fu, Zhiguo Wang, Jingquan Zhu, Liang Ma

**Affiliations:** ^1^ Institute of Soil Fertilizer, Agricultural Water Saving, Xinjiang Academy of Agricultural Sciences, Urumqi, China; ^2^ Xinjiang Academy of Agricultural Sciences, Baicheng Agricultural Experimental Station/National Soil Quality Aksu Observation Experimental Station, Aksu, China; ^3^ Scientific and Technological Achievement Transformation Center, Xinjiang Academy of Agricultural Sciences, Urumqi, China; ^4^ College of Hydraulic and Civil Engineering, Xinjiang Agricultural University, Urumqi, China

**Keywords:** micro-/nanobubble oxygenation irrigation, maize, rhizosphere, microbial community, available phosphorus, yield

## Abstract

Micro-/nanobubble oxygenation irrigation, as a novel irrigation technique, has been widely utilized to enhance soil phosphorus availability and maize yield. Nevertheless, currently, most of the studies remain unclear about the precise mechanism through which micro-/nanobubble oxygenation improves soil phosphorus availability and maize yield. Therefore, we established two irrigation methods, conventional irrigation (CF) and micro-/nanobubble oxygenation irrigation (MB), to investigate the combined effects on enzyme activity, microbial communities, and soil phosphorus availability in the rhizosphere soil of maize.The results showed that compared to the CF treatment, the MB treatment significantly increased available phosphorus content and alkaline phosphatase activity in maize rhizosphere soil by 21.3% and 15.4%, respectively. Furthermore, MB significantly influenced bacterial diversity in the maize rhizosphere soil but did not considerably affect fungal diversity. Specifically, MB regulated the microbial community structure in the maize rhizosphere by altering the relative abundances of the bacterial phylum Firmicutes and the fungal phyla Mucoromycota, Chytridiomycota, and Basidiomycota. In addition, MB reduced the complexity of the bacterial network while increasing the interaction density among bacterial species. Meanwhile, MB enhanced the complexity of the fungal network. Structural equation modeling indicated that MB primarily promoted soil alkaline phosphatase activity by regulating bacterial community diversity, thereby enhancing soil phosphorus availability. In conclusion, the application of micro-/nanobubble oxygenation irrigation enhances the activity of alkaline phosphatasein the soil by modulating the microbial community within the rhizosphere, thereby facilitating increased phosphorus availability in the rhizosphere of maize.

## 1 Introduction

Xinjiang, located in northwestern China, is characterized as a typical arid region and serves as a significant grain production base within the country, with maize (*Zea mays* L.) being the predominant grain crop (Erenstein et al., 2021; Muthuvel et al., 2023). The region’s low precipitation levels and high rates of evapotranspiration, combined with the requirement for prolonged irrigation, have contributed to a rise in the water table, leading to considerable secondary soil salinization (Wang et al., 2022). This has led to a notable reduction in arable land and a decline in grain production, which has severely constrained the development of local agriculture. The process of soil salinization has been demonstrated to result in a reduction in microbial diversity and a subsequent decline in soil fertility, which in turn gives rise to deficiencies in crop nutrients. This not only leads to crop growth inhibitory but also compromises the functionality of the soil ecosystem (Ren et al., 2023). Phosphorus (P) is a vital nutrient for plant growth. However, the uptake of phosphorus by crop roots is frequently constrained by competition from salt ions, such as Na^+^ and Cl^−^, which result in low phosphorus effectiveness (Jiang et al., 2023). In recent years, the application of phosphorus fertilizers has significantly improved maize yields in Xinjiang, particularly through the widespread use of chemical phosphorus fertilizers. Although these fertilizers effectively alleviate the negative impacts of saline soils on crop productivity, their usage remains suboptimal (Guo et al., 2023; Li and Li, 2022; Ma et al., 2023). Therefore, there is an urgent need to develop effective strategies to enhance phosphorus availability and ensure food production security.

Oxygen (O) is a vital component for the aerobic respiration of numerous microorganisms. An increase in O concentration stimulates the metabolic activity of soil microorganisms, thereby enhancing the overall activity of soil microorganisms (Li Z. et al., 2024). This alteration impacts not only microbial diversity but also the ecological function of the soil and, in many cases, the efficacy of nutrients in the soil. Microbial communities play a pivotal and multifaceted role in regulating soil phosphorus effectiveness. A multitude of microorganisms, including bacteria and fungi, are capable of solubilizing mineral phosphates and converting soil P into forms that are more readily bioavailable to plants (Zhang et al., 2023; Chen X. D. et al., 2019; Jiao et al., 2021). Concurrently, crop roots secrete metabolites, such as phosphatases, which facilitate the conversion of soil phosphorus components that are difficult to utilize into a form that can be absorbed by the crop (Li S. et al., 2024). Micro- and nanobubbles (MB) are defined as bubbles with particle sizes ranging from 200 nm to 50 μm situated between micro- and nanobubbles. Due to their small particle size, large specific surface area, and high pressure, micro-/nanobubbles are characterized by a long storage time, strong aerosolization, and adsorption (Liu et al., 2019). The application of micro-/nanobubble oxygenation irrigation has been demonstrated to enhance the structure of the soil microbial community, stimulate soil respiration, and augment the abundance of soil microorganisms, thereby improving soil fertility and nutrient uptake (Chen H. et al., 2019; Zhu et al., 2019; Yu et al., 2022; Zhao et al., 2023). The findings of several studies have indicated that micro-/nanobubble oxygenation can facilitate the effective conversion of organic phosphorus in soil, thereby providing crops with a greater availability of phosphorus resources (Nishigaki et al., 2021; Wang et al., 2023). Nevertheless, the precise mechanism by which micro-/nanobubble oxygenation affects interroot soil microbial and enzyme activities, thereby enhancing soil phosphorus effectiveness, remains unclear.

In this study, field soil column cultivation was employed to investigate the effects of conventional flood irrigation (CF) and MB on available phosphorus, alkaline phosphatase (ALP) activity, and microbial community diversity and structure in maize rhizosphere soil. The objectives of this study were 1) to evaluate the response of phosphorus availability, ALP activity, and microbial communities in maize rhizosphere soil to MB oxygenation irrigation; 2) to explore the interactions among rhizosphere soil phosphorus availability, phosphatase activity, and microbial communities; and 3) to clarify the key factors for improving soil phosphorus efficiency and crop yield by MB oxygenation irrigation.

## 2 Materials and methods

### 2.1 Experimental site

**Figure 1:**
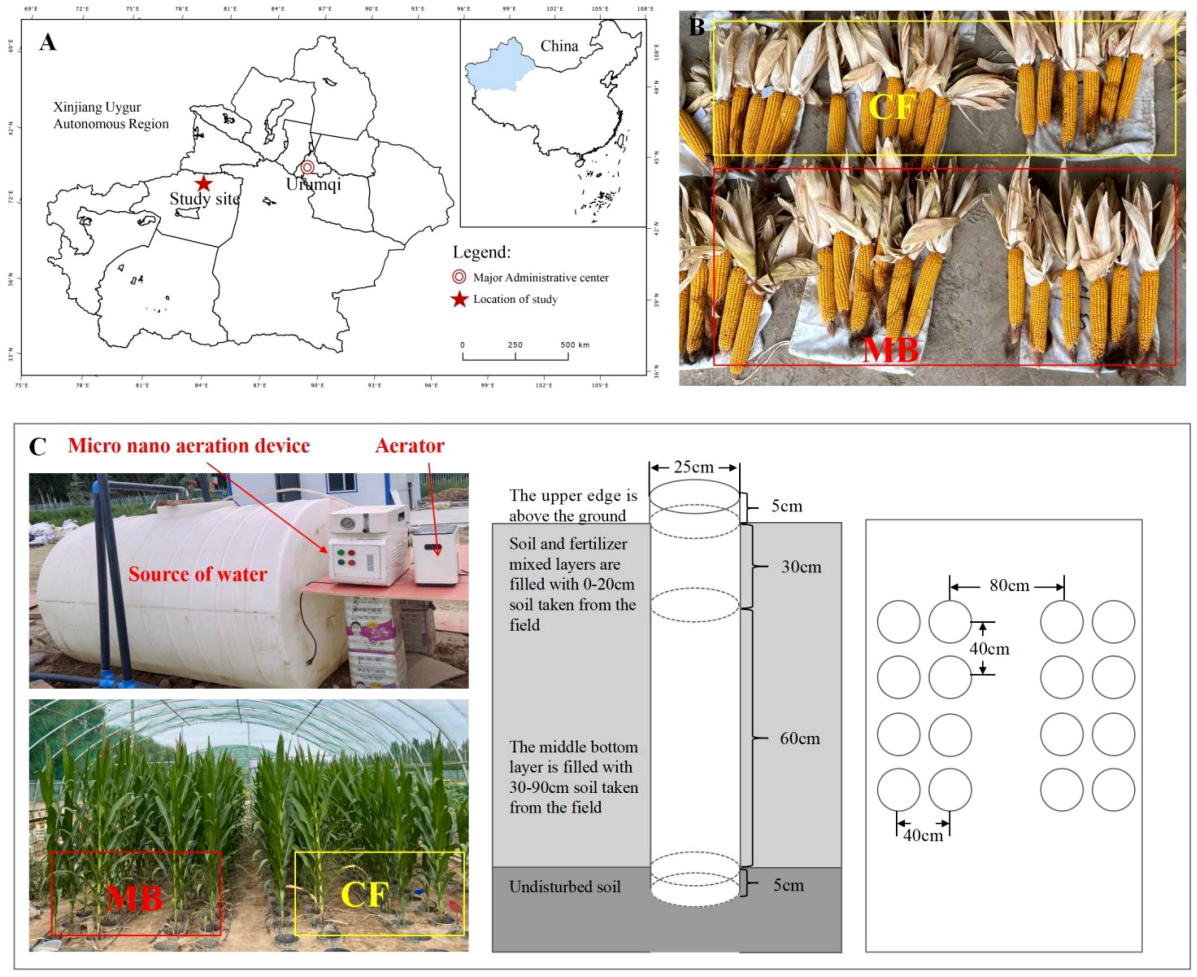
Experimental location and schematic diagram of the experimental setup. **(A)** The location and layout of the study area; **(B)** a photograph of the yield. **(C)** A schematic diagram of the experimental design. The experimental site was located in the southern region of Xinjiang, China. The experiment was conducted mainly in field soil column cultivation, and the irrigation equipment mainly consisted of three parts: micro-/nanobubble generator, oxygen supply device, and water source; the irrigation methods were set up in two ways: conventional irrigation (CF) and micro-/nanobubble oxygenation irrigation (MB).

The experiment was conducted in 2023 at the National Soil Quality Aksu Observation and Experiment Station, located in Baicheng County, Aksu Prefecture, Xinjiang, with the following geographic coordinates: 81°92′E, 41°80′N. The specific location is shown in **Figure 1A**. The test soil was collected from the National Soil Quality Aksu Observation and Experiment Station, and the soil type is brown desert soil. The soil texture is sandy loam with moderate fertility. The climate characteristics of Baicheng County (project area) in Xinjiang belong to a temperate continental arid climate, with an annual average temperature of 7.6°C, an extreme max temperature of 38.3°C, an extreme min temperature of −28°C, a frost-free period of 133–163 days, an annual average sunshine duration of 2,789.7 h, and an annual average precipitation of 171.13 mm. The physicochemical properties of the rhizosphere soil are shown in **Table 1**.

**Table 1:** Physicochemical properties of rhizosphere soil.

pH	Water-soluble salt (g·kg^−1^)	Hydrolyzable nitrogen (mg·kg^−1^)	Organic phosphorus (mg·kg^−1^)	Available phosphorus (mg·kg^−1^)	Potassium (mg·kg^−1^)	Organic matter (g·kg^−1^)	Total nitrogen (g·kg^−1^)	Carbon–nitrogen ratio (%)
8.12 ± 0.04	3.1 ± 0.1	107 ± 6	278.4 ± 10.1	14.7 ± 0.8	409 ± 9	27.2 ± 0.7	1.68 ± 0.05	16.17 ± 0.09

Data are presented as mean ± standard deviation (SD) based on three replicates.

### 2.2 Experimental design

The test soil was collected from the National Soil Quality Aksu Observation and Experiment Station, which had not been applied with any fertilizer for the past 5 years. The soil type is brown desert soil with a silty (sandy) loam texture. Soil samples were collected from the 0–20-cm plow layer and the 20–90-cm subsoil layer. These samples were subsequently air-dried, sieved through a 1-cm mesh, homogenized, and reserved for further analysis. Polyvinyl chloride (PVC) pipes, each possessing an internal diameter of 25 cm and a height of 100 cm, were installed within the soil substrate. The uppermost 5 cm of these pipes extended above the soil surface to mitigate the occurrence of surface runoff following precipitation events. The bottom of the pipes was left open to ensure direct contact with the natural soil, simulating natural field conditions. Each soil column was filled with 50 kg of dry soil, divided into two layers: 30–90 cm of subsoil from the field and 0–30 cm of plow layer soil from the field. After mixing with fertilizer, the soil was added to the columns, and water was applied to compact the soil after each addition (**Figure 1B**).

The experiment was conducted at the National Soil Quality Aksu Observation and Experiment Station using field soil column cultivation. The spring maize variety “Tianyu 303” was used as the test material. Sowing was done on 1 May 2023, and harvesting was on 1 October 2023. Two distinct irrigation techniques were employed: CF and MB. The former was derived from surface water within the project area, exhibiting a dissolved oxygen (DO) concentration of 8–9 mg/L. The micro-/nanobubble oxygenation irrigation was prepared using a B&W micro-/nanobubble generator [produced by Benzhou (Beijing) New Technology Promotion Co., Ltd., Beijing, China with a working pressure of 0.015 MPa and an inlet flow rate of 1.5 L/min]. In order to enhance the DO content in the irrigation water, an oxygen supply device (Jiangsu Yuyue Medical Equipment Co., Ltd., DanYang, China, YU300 type, oxygen flow rate of 2 L/min) was utilized, set to the maximum amount of oxygen supply (oxygen supply concentration of 90%), and connected with the B&W micro-/nanobubble generating device. The B&W micro-/nanobubble generating device was operated to stabilize the changes in dissolved oxygen, and then the HQ40 type was employed. A portable dissolved oxygen meter (Seven2Go™, Mettler Toledo International Trading Co., Ltd., Shanghai, China, ± 0.1 mg/L) was employed to monitor the dissolved oxygen concentration content of the water body (DO = 30 mg/L). The formation of micro-/nanobubble irrigation water was transported to the root zone of the crop through the drip irrigation system. The phosphorus fertilizer used was superphosphate (total phosphorus content ≥ 34%, available phosphorus content ≥ 32%); the application rate was 258 kg hm^−2^, applied as a basal fertilizer before sowing. Nitrogen (N) and potassium (K) fertilizers were applied according to local farming practices (**Figure 1C**).

### 2.3 Measurement and application methods

#### 2.3.1 Sample collection and processing

Numerous research findings showed that microbial diversity increases and the distribution of microbial community in the rhizosphere becomes more uniform during the process of crop maturity (Hu et al., 2020). Therefore, the rhizosphere soil samples were collected at the mature stage (25 September) of maize, and three soil columns were randomly selected for sampling in each treatment. Rectangular soil blocks (20 cm × 20 cm × 40 cm) were cut vertically downward around the maize root system. The loose soil was then removed from the root system by gently shaking it off, and the soil adhering to the root surface was brushed off and placed into a sterile bag. This bag was then transported back to the laboratory in a timely manner. The interroot soil was divided into three portions for subsequent analysis. One portion was air-dried and ground through a sieve to determine the effective phosphorus content. The second portion was placed in an ice box to determine the ALP activity. The third portion was stored in a refrigerator at −80°C for subsequent sequencing of soil microorganisms.

#### 2.3.2 Determination of available phosphorus and alkaline phosphatase activity in rhizosphere soil

The soil samples were allowed to air-dry and then passed through a 1-mm sieve to determine the effective phosphorus content in the interroot soil. The soil in the project area is alkaline and was extracted with sodium bicarbonate. Following leaching with 0.5 mol/L of NaHCO_3_ (pH 8.5), the concentration of phosphorus was determined by the molybdenum–antimony colorimetric method (Cardelli et al., 2019). The alkaline phosphatase activity in the soil was estimated by determining the activity of p-nitrophenyl phosphate (PNPP) released at 410 nm using Tabatabai’s method (Urlić et al., 2023).

#### 2.3.3 PCR amplification and high-throughput sequencing

A fresh rhizosphere soil sample weighing 0.5 g was used, and its microbial DNA was extracted using the E.Z.N.A.^®^ Soil DNA Kit (Omega Bio-tek, Norcross, GA, USA). DNA quality and quantity were determined using a NanoDrop 2000 spectrophotometer (Bio-Rad Laboratories Inc., USA).

Primers 338F (5′-ACTCCTACGGGAGGCAGCA-3′) and 806R (5′-GGACTACHVGGGTWTCTAAT-3′) were used to target the V3–V4 regions, and primers ITS5F (5′-GGAAGTAAAAGT of 16S rRNA in rhizosphere soil bacteria) and ITS1R (5′-GCTGCGTTCTTCATCGATGC-3′) amplified the ITS1 region of the fungus by PCR with the following amplification conditions (25 µL): reaction buffer (5×) 5 µL, GC buffer (5×) 4 µL, 2.5 mM dNTP 2 µL, forward and reverse primers (10 mM) 1 µL each, DNA template 2 µL, Q5 DNA HiFi polymerase 0.25 µL, and ddH_2_O 20 µL. The amplification procedure was as follows: predenaturation at 95°C for 2 min, denaturation at 98°C for 15 s, annealing at 55°C for 30 s, extension at 72°C for 30 s, 25–30 cycles, and finally extension at 72°C for 5 min. The amplification products were sequenced using MiSeq PE300 from Illumina. Using the fastp (Chen et al., 2018) (https://github.com/OpenGene/fastp, version 0.20.0) software, quality control was carried out on the original sequencing sequence. Using the FLASH (Magoč and Salzberg, 2011) (http://www.cbcb.umd.edu/software/flash, version 1.2.7) software, the degree of overlap between reads spliced into tags was determined. These raw labels were then filtered using the Trimmomatic software (version 0.33) to obtain high-quality labels. Using the UPARSE software (Edgar, 2013) (http://drive5.com/uparse/, version 7.1), operational taxonomic units (OTUs) were clustered with a 97% similarity cutoff (Stackebrandt and Goebel, 1994), optimizing the extraction of repeat sequences and the removal of no single sequence repeat. According to 97% similarity, OTU was used to cluster non-repetitive sequences (excluding single sequences), and the Mosaic was removed in the clustering process to obtain the OTU representative sequence. All optimized sequences were mapped to the OTU representative sequences, and sequences that were more than 97% similar to the OTU representative sequences were selected. Analysis of microbial diversity and community composition was performed after each sample of bacteria and fungi.

#### 2.3.4 Determination of maize grain yield and yield components

Three maize plants from each treatment were selected for indoor yield component analysis. After natural air drying to a grain moisture content of approximately 12.5%, yield and its components were determined (Zhang et al., 2021). The number of ears per plant, the number of rows per ear, and the number of kernels per row were measured. The total weight and 100-kernel weight were recorded after threshing.

### 2.4 Statistical analysis

All data were preprocessed using Excel 2016 (Microsoft, Redmond, USA), SPSS Statistics 27 (IBM, Armonk, USA), and Origin 2021 (OriginLab, Northampton, USA). The experimental area was mapped using ArcGIS software. Boxplots were used to illustrate changes in available phosphorus, soil alkaline phosphatase activity, and microbial community diversity indices across different treatments. Stacked bar plots were employed to display microbial community composition. Differences in community composition between samples from different treatments were examined using differential bubble plots and linear discriminant analysis (LDA). The similarity percentage (SIMPER) analysis, typically based on the Bray–Curtis dissimilarity matrix, was used to identify species contributing to the differences between treatments, quantifying the contribution of each species. Overall differences between treatments were assessed using analysis of similarities (ANOSIM).

To explore the correlations among soil available phosphorus, soil alkaline phosphatase activity, microbial community diversity, and differential microorganisms, Spearman correlation analysis was conducted using Origin 2021 software. Microbial co-occurrence networks were visualized using R version 4.1.1. The partial least squares path modeling (PLS-PM) was performed using the “vegan” and “plspm” packages in R to elucidate the mechanisms by which micro-/nanobubble oxygenation irrigation influences phosphorus availability in rhizosphere soil.

## 3 Results

### 3.1 Maize grain yield and yield components

MB significantly affects the content of maize grain yield and yield components (**Table 2**). The number of rows of kernels, number of kernels per row, number of kernels per spike, dry weight of grain, 100-kernel weight, and yield were 22.2%, 8.8%, 30.0%, 23.8%, 28.2%, and 18.7% higher in the MB treatment than in the CF treatment, respectively.

**Table 2:** Effects of different irrigation methods on maize grain yield and yield components.

Treatments	Kernel row number	Kernels per row	Kernel number per ear	Grain dry weight (g)	Hundred-kernel weight (g)	Yield (kg·hm^−2^)
CF	15.33 ± 1.63	36.33 ± 2.32	522.66 ± 94.04	159.02 ± 13.13	27.42 ± 1.50	15,118.5 ± 17.25
MB	18.73 ± 1.26	39.52 ± 2.69	679.33 ± 60.18	196.81 ± 19.46 b	35.16 ± 2.50	17,942.72 ± 10.56
*p*	*	*	**	**	**	**

Data are presented as mean ± standard deviation (SD) based on three replicates. * and ** indicate significant differences at the *p <*0.05 and *p <*0.01 levels, respectively, and ns indicates no significant difference.

### 3.2 Available phosphorus content and alkaline phosphatase activity

**Figure 2:**
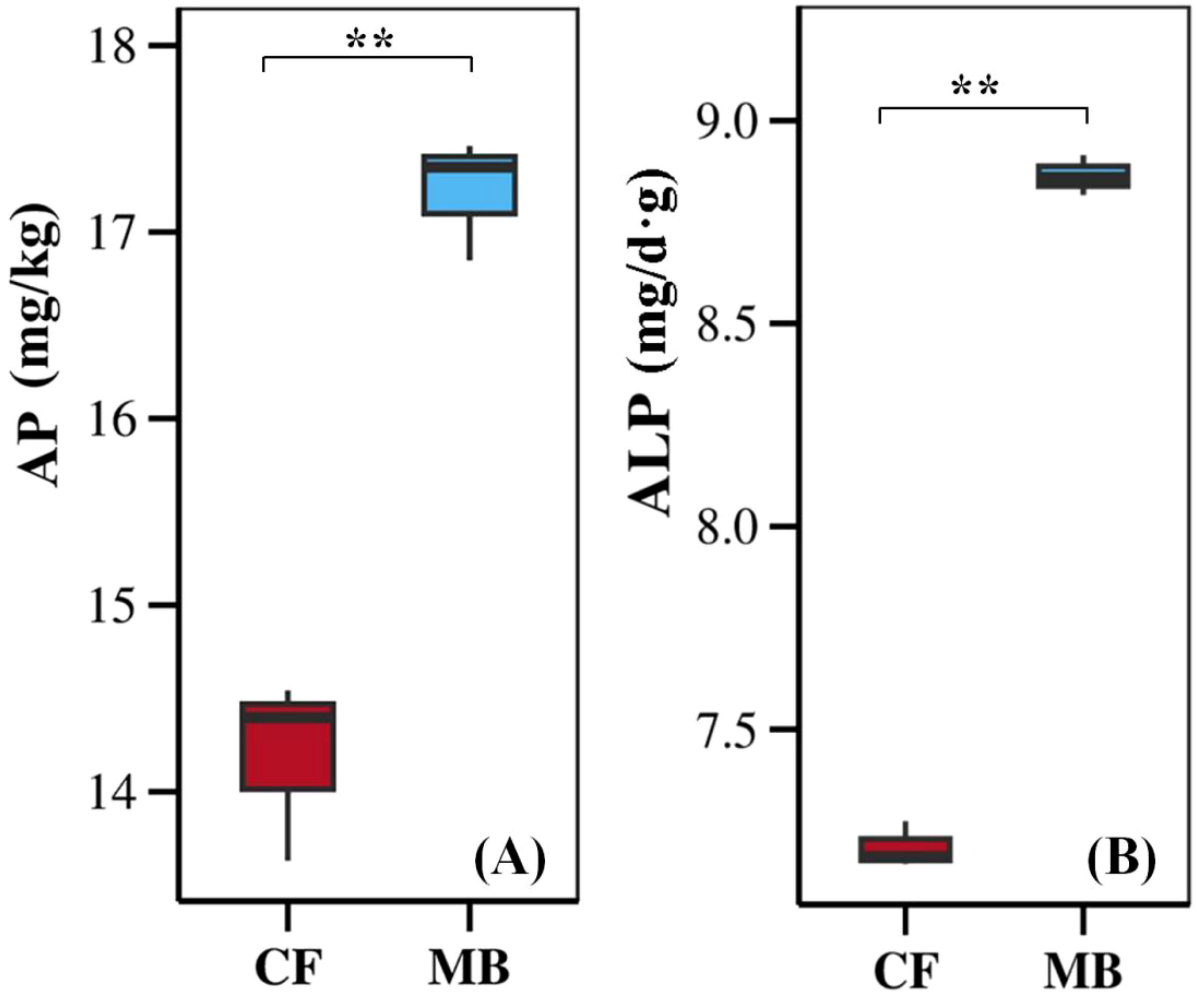
Effects of different irrigation methods on available phosphorus content **(A)** and soil alkaline phosphatase activity **(B)** in rhizosphere soil. The boxplots display the data for each treatment, with the boxes representing the interquartile range (IQR), the horizontal line inside the box indicating the median, and the whiskers extending to 1.5 times the IQR. ** indicate significant differences at the and *p <*0.01 levels, respectively, and ns indicates no significant difference. AP, available phosphorus; ALP, alkaline phosphatase; CF, conventional flood irrigation; MB, micro-/nanobubble oxygenation irrigation.

MB significantly affects the content of available phosphorus and the activity of soil alkaline phosphatases in the rhizosphere soil (**Figure 2**). The available phosphorus content and soil alkaline phosphatase activity in the rhizosphere soil under the MB treatment were increased by 21.3% and 15.4%, respectively, compared to CF.

### 3.3 Microbial community diversity

**Figure 3:**
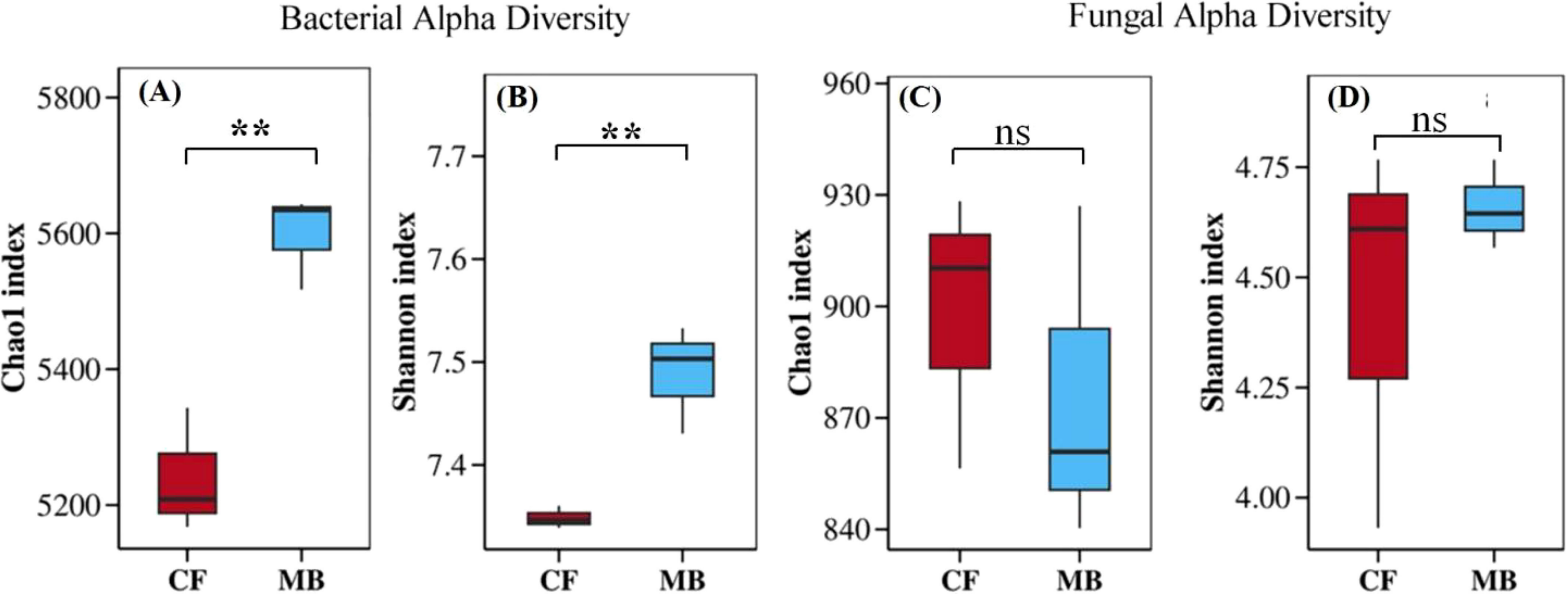
Effects of different irrigation methods on microbial community diversity in rhizosphere soil. Bacterial alpha diversity is represented by the Chao1 index **(A)** and the Shannon index **(B)**. Similarly, fungal alpha diversity is represented by the Chao1 index **(C)** and the Shannon index **(D)**. The boxplots show data for each treatment, with the boxes representing the interquartile range (IQR), the horizontal line inside the box indicating the median, and the whiskers extending to 1.5 times the IQR. ** indicate significant differences at the and *p <*0.01 levels, respectively, and ns indicates no significant difference. CF, conventional flood irrigation; MB, micro-/nanobubble oxygenation irrigation.

MB significantly affects the microbial community diversity in the rhizosphere soil. The Chao1 index and the Shannon index of rhizosphere soil bacteria under the MB treatment increased by 6.8% and 1.9%, respectively, compared to the CF treatment (**Figures 3A, B**). However, there were no significant differences in the Chao1 index and the Shannon index of rhizosphere fungi between the CF and MB treatments (**Figures 3C, D**). Overall, MB had a significant impact on bacterial diversity in the rhizosphere soil but did not significantly affect fungal diversity in the rhizosphere soil.

### 3.4 Microbial community structure

**Figure 4:**
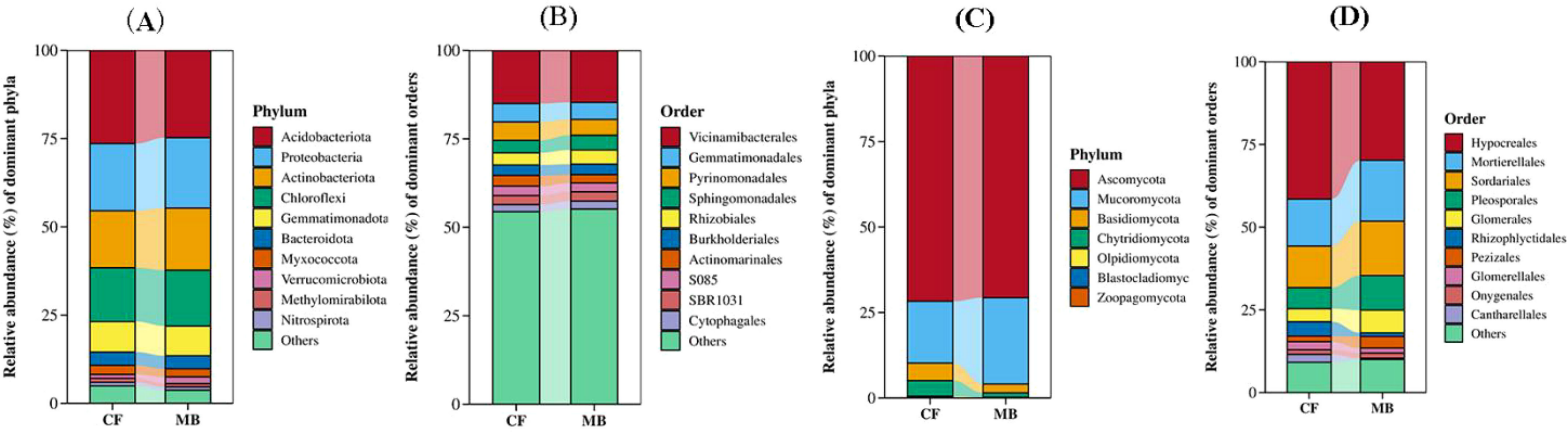
Effects of different irrigation methods on rhizosphere soil microbial community structure. **(A)** Bacterial community structure at the phylum level (top 10). **(B)** Bacterial community structure at the order level (top 10). **(C)** Fungal community structure at the phylum level (top 10). **(D)** Fungal community structure at the class level (top 10). CF, conventional flood irrigation; MB, micro-/nanobubble oxygenation irrigation.

In the rhizosphere soil samples obtained from various treatments, the 10 most abundant species were selected for presentation, with all remaining species categorized under the designation “Others.” The phylum-level analysis of bacterial community composition showed the following dominant phyla (relative abundance > 5%) in the tested rhizosphere soil: Acidobacteriota, which was the most abundant, followed by Proteobacteria, Actinobacteriota, Chloroflexi, and Gemmatimonadota. There were no significant differences in the relative abundances of these phyla between the CF and MB treatments (**Figure 4A**). At the order level, the dominant orders (relative abundance > 5%) in the tested rhizosphere soil were Vicinamibacterales, which was the most abundant, followed by Gemmatimonadales. The relative abundances of these orders did not differ significantly between the CF and MB treatments (**Figure 4B**).

The phylum-level analysis of fungal community composition revealed that Ascomycota exhibited the highest abundance (average abundance 71.19%) in the rhizosphere soil, with no statistically significant differences observed between the CF and MB treatments. Mucoromycota was the second most abundant (average abundance 21.67%), and its relative abundance was significantly increased by 39.79% under MB compared to the CF treatment (**Figure 4C**). At the order level, Hypocreales was the most abundant, followed by Mortierellales, Sordariales, Pleosporales, and Glomerales. The relative abundance of Hypocreales was significantly decreased by 28.27% under MB compared to the CF treatment, while the relative abundances of Hypocreales, Mortierellales, Sordariales, Pleosporales, and Glomerales were significantly increased by 30.04%, 30.21%, 65.34%, and 74.37%, respectively, under MB compared to the CF treatment (**Figure 4D**).

### 3.5 Linear discriminant analysis

**Figure 5:**
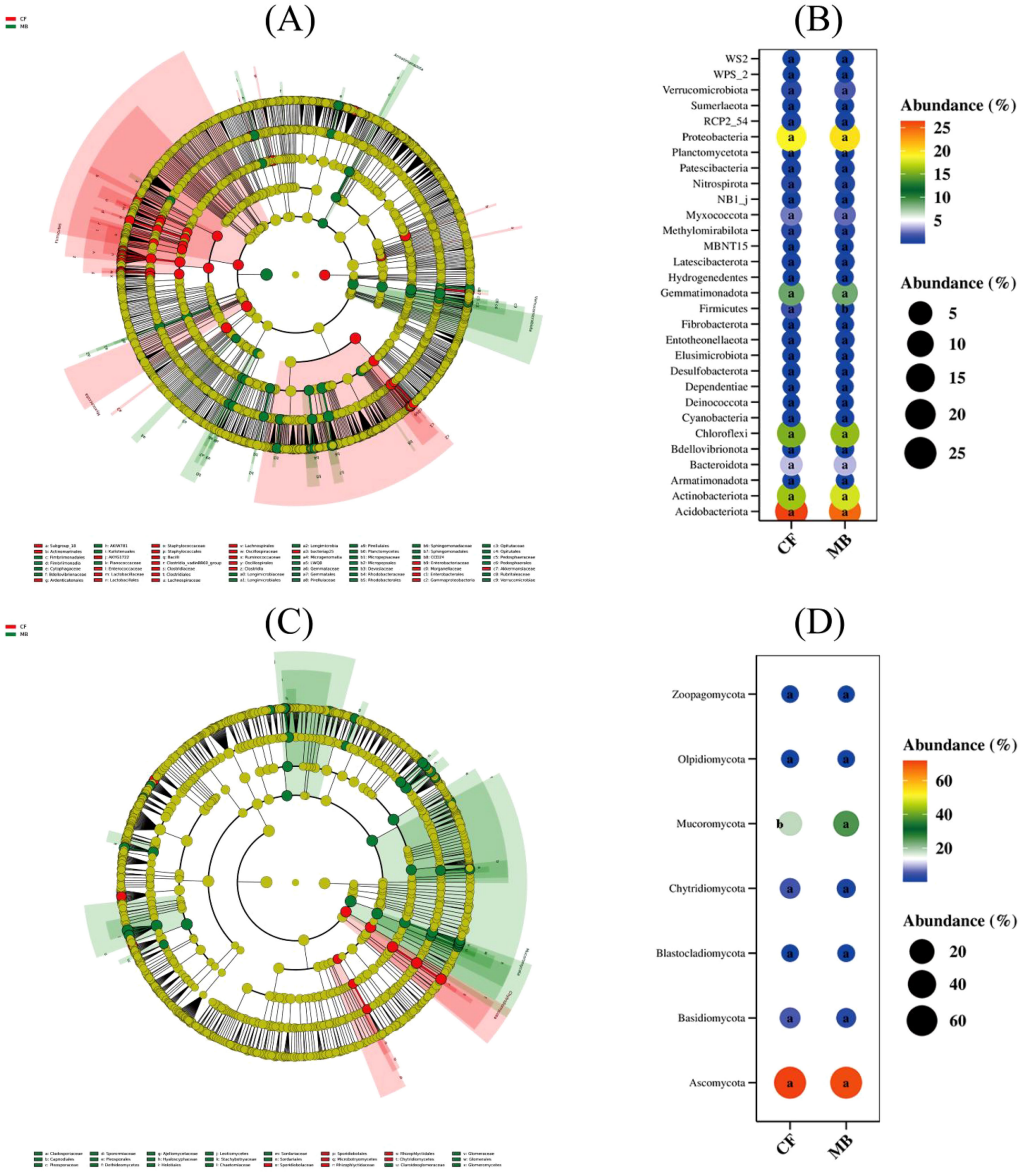
Effects of different irrigation methods on rhizosphere soil microbial community structure. **(A)** Differences in bacterial community structure at the phylum level. **(B)** Differences in bacterial community structure at the order level. **(C)** Differences in fungal community structure at the phylum level. **(D)** Differences in fungal community structure at the class level. CF, conventional flood irrigation; MB, micro-/nanobubble oxygenation irrigation.

LDA effect size analysis indicated significant differences between treatments for 42 bacterial groups (**Figure 5A**) and 21 fungal groups (**Figure 5C**) in the rhizosphere soil. Among the 42 bacterial groups, the largest difference between treatments was observed in the phylum Firmicutes. Among the 21 fungal groups, the largest differences were observed in the phyla Mucoromycota, Chytridiomycota, and Basidiomycota. Differential species bubble plots confirmed significant differences between the MB and CF treatments for Firmicutes (**Figure 5B**), Mucoromycota (**Figure 5D**), and Chytridiomycota (**Figure 5D**).

### 3.6 Similarity percentage analysis

SIMPER analysis of differential contribution showed that in the rhizosphere soil bacterial community (**Table 3**), the microbial species with high contribution rates (Ava, Avb) in both the conventional flood irrigation (CF) and micro-/nanobubble oxygenation irrigation (MB) treatments were Acidobacteriota, Actinobacteriota, Proteobacteria, Chloroflexi, and Gemmatimonadota, but no significant differences were observed between treatments. The contribution rates of Firmicutes under the CF and MB treatments were 1.59% and 0.27%, respectively, with a significant difference, and Firmicutes contributed the most to the overall difference (ratio = 5.75), making it the main factor causing differences in microbial composition between the CF and MB treatments.

**Table 3:** Contribution of micro-/nanobubble oxygenation irrigation to differences in rhizosphere soil bacterial and fungal community structures.

Microbial species		Ratio	Ava (%)	Avb (%)	*p*
Bacterial	Acidobacteriota	1.39	26.42	24.73	>0.01
	Actinobacteriota	1.37	16.15	17.61	>0.01
	Proteobacteria	1.21	19.03	19.95	>0.01
	Firmicutes	5.75	1.59	0.27	<0.01
	Chloroflexi	1.37	15.22	15.86	>0.01
	Verrucomicrobiota	2.05	1.15	1.91	>0.01
	Gemmatimonadota	1.16	8.65	8.45	>0.01
	Bacteroidota	2.60	3.74	3.64	>0.01
	Myxococcota	1.93	2.59	2.52	>0.01
	Methylomirabilota	1.64	1.08	0.95	>0.01
Fungal	Ascomycota	1.80	71.72	70.65	>0.01
	Mucoromycota	3.20	18.09	25.27	<0.01
	Chytridiomycota	2.97	4.57	1.25	<0.01
	Basidiomycota	2.61	5.09	2.69	<0.01
	Olpidiomycota	1.41	0.34	0.28	>0.01
	Blastocladiomycota	1.13	0.1	0.16	>0.01
	Zoopagomycota	0.69	0.08	0.02	>0.01

The ratio represents the proportion of each microbial variable’s contribution to the overall difference. “Ava” indicates the contribution rate of the microbial variable in the conventional flood irrigation treatment, while “Avb” denotes the contribution rate in the micro-/nanobubble oxygenation irrigation treatment. Statistical significance is indicated by *p*-values at the 0.01 level. The top 10 bacterial species based on their contribution to the soil bacterial community structure were selected, whereas the contribution of fungal species in the soil fungal community structure is concentrated among these key species.

In the rhizosphere soil fungal community, Ascomycota had the highest contribution rates in both the CF and MB treatments, with no significant difference between treatments. Mucoromycota, Chytridiomycota, and Basidiomycota showed significant differences between treatments, and these three fungal phyla contributed significantly more to the overall differences compared to other fungal species.

### 3.7 Soil microbial co-occurrence networks

**Figure 6:**
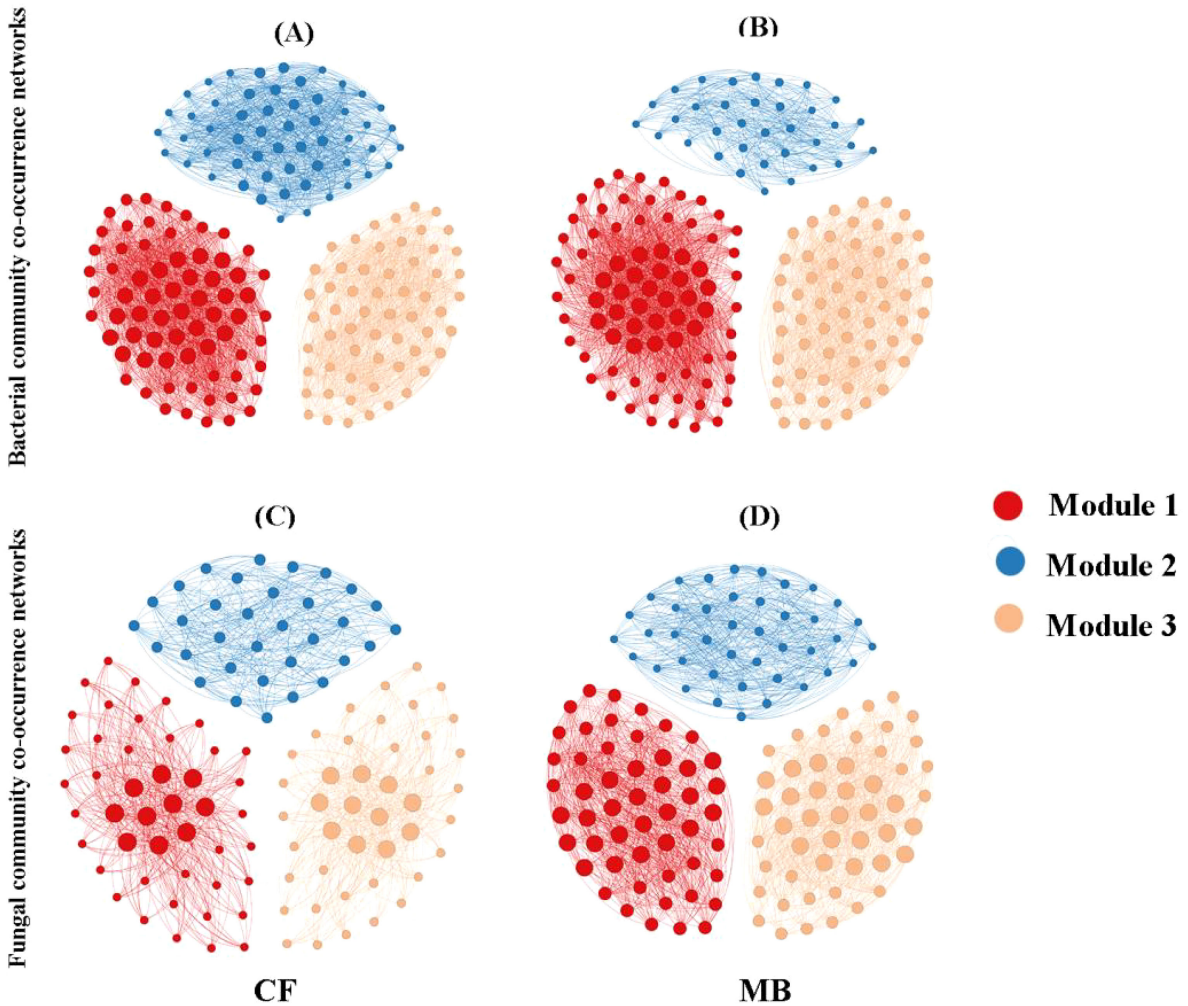
Symbiotic networks of bacterial **(A, B)** and fungal **(C, D)** communities in rhizosphere soil under different irrigation treatments. Nodes represent operational taxonomic units (OTUs). Modules are color-coded to indicate different network clusters. CF, conventional flood irrigation; MB, micro-/nanobubble oxygenation irrigation.

To elucidate the variations in soil microbial network interactions among distinct bacterial taxa, co-occurrence networks were constructed utilizing OTUs (**Figure 6**). Based on the calculation of the topological characteristics of bacterial and fungal networks (**Table 4**), the nodes in these networks were allocated into three modules. In the microbial network diagram, modules with the same color represent groups of microbial taxa that may share similar phylogenetic and interaction characteristics and possibly similar ecological niches.

**Table 4:** Topological characteristics of co-occurrence networks for different taxa in rhizosphere soil bacterial and fungal communities.

Network parameters	Bacterial		Fungal	
	CF	MB	CF	MB
Nodes	182	172	121	142
Edges	5,484	5,291	2,421	3,320
Average degree	60.26	61.52	40.02	46.76
Diameter	1	1	1	1
Density	0.33	0.36	0.33	0.34
Clustering coefficient	1	1	1	1
Average path length	1	1	1	1

CF, conventional flood irrigation; MB, micro-/nanobubble oxygenation irrigation.

The bacterial network in the MB treatment exhibited a reduction in nodes and edges compared to that in the CF treatment while simultaneously increasing the average degree and density. The diameter, clustering coefficient, and average path length of the bacterial networks were similar between the MB and CF treatments. Overall, the reduction in nodes and edges in the MB treatment bacterial network weakened the overall complexity of the network, but the increase in average degree and density strengthened the connections and interactions between microbes, leading to a denser relationship among bacterial species. For the fungal network, in comparison to the CF treatment, the MB treatment increased nodes, edges, average degree, and density, while the diameter, clustering coefficient, and average path length were similar between the MB and CF treatments. This indicates that the MB network is more complex.

### 3.8 Correlations among rhizosphere soil available phosphorus, soil phosphatase activity, and soil microbial diversity

Based on the LDA effect size analysis, the greatest differences among bacterial taxa between treatments were observed in the phylum Firmicutes, while the largest differences in fungal taxa were found in Mucoromycota and Chytridiomycota. Spearman correlation analysis was primarily performed on the differential species.

**Figure 7:**
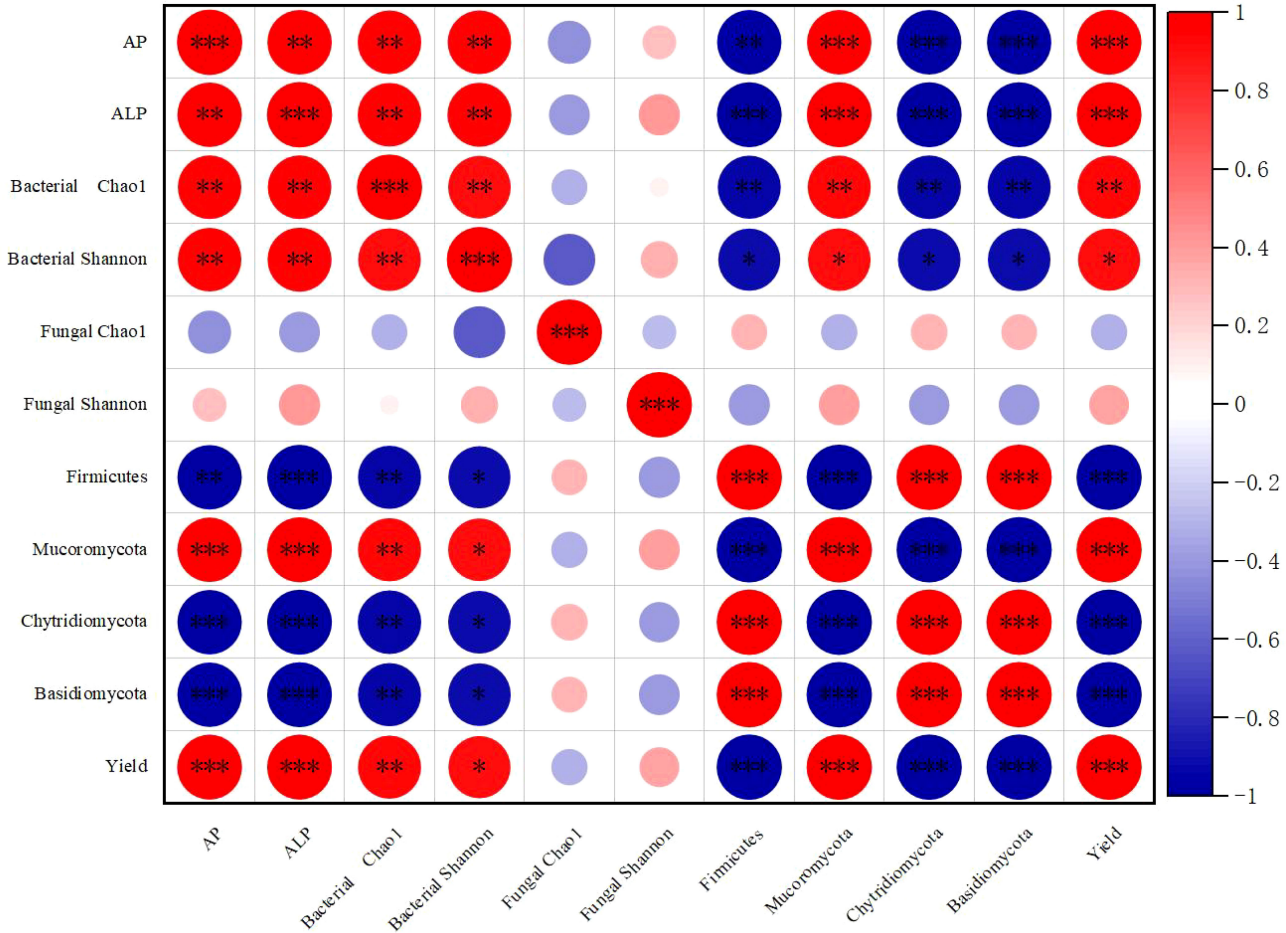
Spearman correlation among rhizosphere soil available phosphorus, soil phosphatase activity, and soil microbial community. The area of the bubble represents the absolute value of the phase relation matrix, and the color of the bubble represents the correlation coefficient. *, **, *** indicate significant differences at the p < 0.05, p < 0.01 and p < 0.001 levels respectively.

Spearman correlation analysis (**Figure 7**) revealed significant positive correlations between rhizosphere soil available phosphorus and soil phosphatase activity, bacterial diversity (Chao1 index and Shannon index), and the relative abundance of Mucoromycota. Conversely, it showed significant negative correlations with the phyla Firmicutes, Mucoromycota, and Chytridiomycota. Additionally, rhizosphere soil available phosphorus had a negative correlation with the fungal community Chao1 index and a positive but weaker correlation with the fungal community Shannon index.

### 3.9 Structural equation model

**Figure 8:**
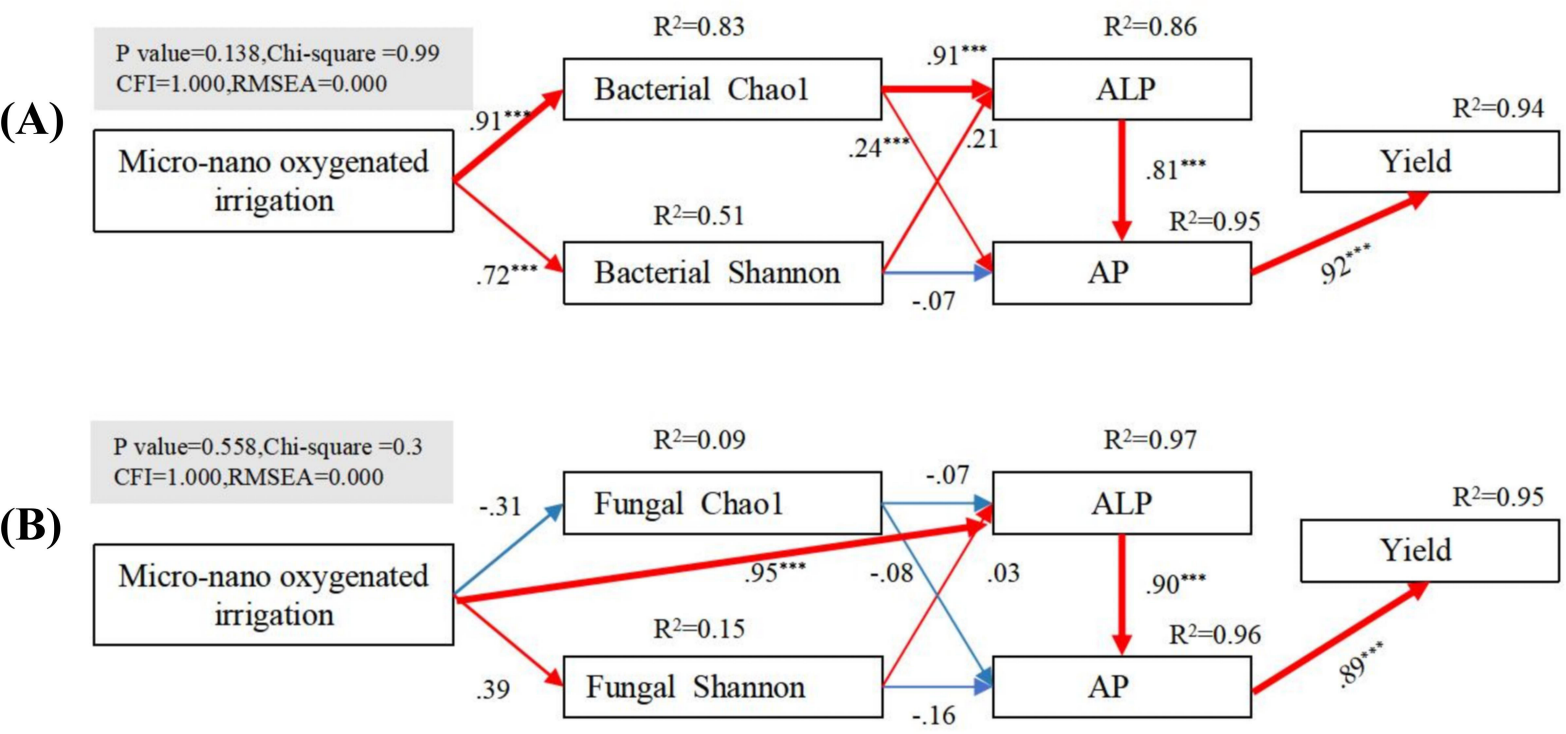
Structural equation modeling (SEM) analysis of the effects of micro-/nanobubble oxygenation irrigation on rhizosphere soil bacterial **(A)** and fungal **(B)** diversity, available phosphorus, and phosphatase activity, and maize yield. The figure shows the explained variance (*R*²) for each parameter in the model. Red and blue solid lines represent significant positive and negative correlations, respectively (***p < 0.001).

A structural equation model (SEM) was used to explore the mechanisms by which micro-/nanobubble oxygenation irrigation affects available phosphorus, phosphatase activity, and microbial diversity in the rhizosphere soil. The results showed that micro-/nanobubble oxygenation irrigation significantly influences bacterial community diversity (e.g., bacterial Chao1 and bacterial Shannon), with the strongest effect on bacterial Chao1. Bacterial Chao1 showed the strongest effect on ALP, which responded strongly to available phosphorus (AP), and AP, in turn, showed a strong response to yield (**Figure 8A**). In contrast, fungal diversity (e.g., fungal Chao1 and fungal Shannon) did not show a significant response to oxygenation irrigation (**Figure 8B**). However, ALP exhibited the strongest response to oxygenation irrigation and had a significant effect on AP, which, in turn, responded strongly to yield. The study confirms that micro-/nanobubble oxygenation irrigation mainly enhances soil available phosphorus and yield by regulating bacterial diversity and increasing ALP.

## 4 Discussion

ALP plays a crucial role in the mineralization of organic phosphorus compounds in alkaline soils. ALP is primarily synthesized by microorganisms, as plant roots are generally not considered to release ALP (Wang et al., 2021). Therefore, ALP activity is one of the enzymes involved in microbial-mediated mineralization of soil organic phosphorus (Chen et al., 2023). However, most of the current studies are limited to the following measures to regulate alkaline phosphatase activity: cropping patterns (An et al., 2024; Qu et al., 2024), conditioners (Hu et al., 2023; Xing et al., 2024), tillage practices (Zhang et al., 2023), and exogenous nutrient inputs (Garg and Bahl, 2008; Cao et al., 2022). The research on how to utilize irrigation technological tools to regulate soil enzyme activities is still limited. In this study, MB increased soil alkaline phosphatase activity by 15.4% compared to CF, which further illustrates the potential of micro-/nanobubble oxygenation irrigation technology in enhancing the activity of key enzymes of phosphorus cycling and is well validated (Li et al., 2016; Zhao et al., 2023).

Microorganisms play a significant role in the mineralization of organic phosphorus and the solubilization of inorganic phosphorus, which can contribute to an increase in soil-effective phosphorus (AP) content. The oxygen requirements of microorganisms vary, with an increase in oxygen leading to a relative increase in aerobic microorganisms and a potential decrease in anaerobic microorganisms. This can result in significant alterations to the diversity and composition of microbial communities (Sennett et al., 2024). The structural equation modeling analysis conducted in this study concluded that micro-/nanobubble oxygenation irrigation increased soil phosphatase activity, primarily through the modulation of bacterial community diversity, thereby enhancing soil phosphorus effectiveness and maize yield. This finding is in accordance with the conclusion of this study, which determined that the impact of oxygenated irrigation on bacterial community diversity was more pronounced than that on fungal community diversity (**Figure 3**). However, it has been demonstrated that oxygenation primarily influences the structure of soil fungal communities and stimulates crop growth (Li et al., 2023). This may be attributed to the fact that the impact of micro-/nanobubble oxygenation irrigation on soil microbial communities is contingent upon a multitude of factors, including the physiological attributes of microorganisms, soil physicochemical properties, crop species, and environmental conditions (Li et al., 2016). Furthermore, in conjunction with LDA and SIMPER difference contribution analysis, it was initially determined that oxygenated irrigation primarily increased the abundance of Mucoromycota and decreased the abundance of Firmicutes, Chytridiomycota, and Basidiomycota. This was achieved by increasing the abundance of Mucoromycota and modifying the microbial community structure, which resulted in a favorable impact on the enhancement of soil-effective phosphorus. The results of the Spearman correlation analysis (**Figure 3**) indicated a positive correlation between interroot soil phosphatase activity, bacterial diversity, and Mucoromycota and a significant negative correlation with Firmicutes, Chytridiomycota, and Basidiomycota. We hypothesized that the increase in alkaline phosphatase activity could be due to the secretion of Micrococcaceae and Nocardioidaceae and changes in the abundance of Mucoromycota, Firmicutes, Chytridiomycota, and Basidiomycota. These phenomena suggest that micro-/nanobubble oxygenation irrigation can promote the mineralization of organic phosphorus in soil and increase yield by changing the structure and diversity of interroot bacterial communities. Jian W concluded that strawberry yield and quality were improved by altering soil bacterial communities (Wang et al., 2023). It has been reported in many previous studies that the abundance of microorganisms in the soil was improved with the presence of micro-/nanobubble oxygenation irrigation, resulting in a more stable and healthy soil ecosystem (Zhang et al., 2024). In this study, micro-/nanobubble oxygenation irrigation altered the structure and diversity of the key interroot microbial community of maize and facilitated the secretion of alkaline phosphatases, which in turn increased the soil phosphorus effectiveness and enhanced maize yield.

## 5 Conclusions

This study demonstrates that micro-/nanobubble oxygenation irrigation effectively increases available phosphorus content and phosphatase activity in the maize rhizosphere soil. This irrigation management significantly impacts bacterial diversity while having minimal effects on fungal diversity. By altering the microbial community structure, micro-/nanobubble oxygenation irrigation enhances the abundance of key microorganisms that play a crucial role in increasing available phosphorus levels. The primary factors influencing soil phosphatase activity are changes in bacterial community diversity and the abundance of core microbial species. Overall, micro-/nanobubble oxygenation irrigation improves phosphorus availability by promoting soil alkaline phosphatase activity and supporting beneficial microbial populations. Thus, this irrigation technique offers a promising approach for regulating soil microbial communities, enhancing soil phosphatase activity, and improving phosphorus availability in the maize rhizosphere.

## Data Availability

The original contributions presented in the study are included in the article/supplementary material. Further inquiries can be directed to the corresponding authors.
